# A case of splenic artery aneurysm and rupture in a patient on a vascular endothelial growth factor inhibitor for renal cell carcinoma

**DOI:** 10.1002/cnr2.1567

**Published:** 2021-10-28

**Authors:** Daniel Tesolin, Amer Alaref, Mohammed F. K. Ibrahim

**Affiliations:** ^1^ Northern Ontario School of Medicine Lakehead University Thunder Bay Ontario P3E 2C6 Canada; ^2^ Diagnostic Imaging Department Thunder Bay Regional Health Sciences Centre Thunder Bay Ontario P7B 6V4 Canada; ^3^ Regional Cancer Care Northwest Thunder Bay Regional Health Sciences Centre Thunder Bay Ontario P7B 6V4 Canada

**Keywords:** aneurysm rupture, arterial aneurysm, metastatic renal cell carcinoma, pazopanib

## Abstract

**Background:**

Pazopanib is a vascular endothelial growth factor inhibitor that is used in the treatment of metastatic renal cell carcinoma. Post market reports demonstrate an increasing awareness of the association of arterial aneurysms and dissections with vascular endothelial growth factor inhibitor use, although, few reports exist for pazopanib.

**Case:**

Here we report a 69‐year‐old patient with minimal cardiovascular risk factors who developed a rupture of a splenic arterial aneurysm after more than 5 years of effective treatment with pazopanib for metastatic renal cell carcinoma.

**Conclusion:**

This case report outlines the necessity to monitor patients while on pazopanib, even when there are minimal risk factors and long periods of tolerance.

## 1 INTRODUCTION

Vascular endothelial growth factor (VEGF) inhibitors represent one of the foundations of treatment for metastatic renal cell carcinoma as is demonstrated by the National Comprehensive Cancer Network guidelines.^1^ The anti‐angiogenesis properties of VEGF inhibitors are employed to inhibit tumor proliferation, however, these same properties may be the cause of various adverse cardiovascular effects that are becoming increasingly documented with use of these inhibitors. The most common adverse effects are hypertension, reduction in ventricular ejection fraction and less commonly, thromboembolic and hemorrhagic events.^2^, ^3^, ^4^

The risks associated with these adverse events can be easily mitigated with close monitoring and cessation of the offending agent, but post‐marketing reviews of VEGF inhibitors have demonstrated that there are potentially rare, life‐threatening adverse effects that require even closer attention. A review of the Japanese Adverse Drug Event Database by Oshima et al. found that out of 16 441 patient treated with VEGF inhibitors, 49 of these patients experienced aortic dissection directly related to VEGF inhibitor use.^5^ A similar study by Cheng et al. reviewed the U.S. Food and Drug Administration Adverse Event Reporting System to identify 240 post‐market reports of arterial aneurysm or dissection associated with VEGF inhibitor with a median onset of 94 days and a fatality rate of 22%.^6^

Cheng et al. have also identified 20 published case reports of patients developing an arterial aneurysm or dissection while on VEGF inhibitors but to our knowledge there are no reports on such adverse events for patients solely treated with pazopanib.^6^

It is important to note instances of these arterial events so clinicians can monitor their patients with appropriate vigilance while on treatment. Here we report an interesting case of a patient who was treated with pazopanib for over 5 years for metastatic renal cell carcinoma before developing a splenic artery aneurysm and subsequent rupture.

## 2 CASE REPORT/CASE PRESENTATION

The patient is a 69‐year‐old female who underwent a right radical nephrectomy 10 years ago, in 2011 for a pT3b N1 M0 grade 3 clear cell carcinoma of the right kidney. Adjuvant sunitinib therapy was initiated and then discontinued after 2 months due to myelosuppression and rash. In February 2013, the patient was found to have recurrence in the right lung upon follow‐up CT imaging. The patient was started on sunitinib and then switched to pazopanib February 2014 due to progression. Pazopanib was held briefly during radical radiotherapy to oligometastatic disease.

The patient developed hypertension a couple of months after starting pazopanib. This was effectively treated with amlodipine. After persistently stable disease, she made a joint decision with her oncologist to discontinue her pazopanib June 2019. She did not experience any other side effects while on pazopanib except for some moderate gastric reflux. Her past medical history is only significant for hypothyroidism and hiatal hernia. She has family history of a father who died in his 60s of a myocardial infarction and sisters with hypertension. She has a substance history of a remote 3 pack year smoking history and occasional alcohol use. Her most recent BMI was calculated to be 24.4.

**FIGURE 1:**
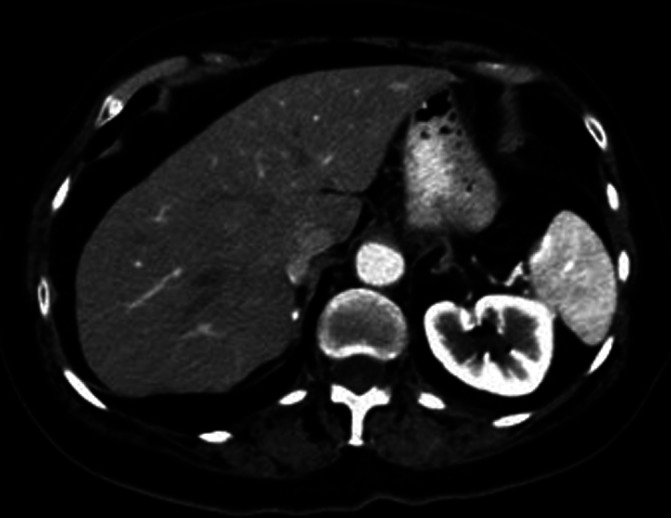
Axial CT SCAN of the abdomen shows no evidence of splenic artery aneurysm. Images acquired February, 2021

In July 2020, CT imaging showed recurrence with enlargement of a left apical pulmonary nodule. She received radical intent radiotherapy with a dose of 60 Gray in 30 fractions, then she was started back on pazopanib. Due to severe reflux symptoms and the development of atrial fibrillation, pazopanib was briefly held January 2021 and restarted February 22, 2021. She was also placed on bisoprolol and warfarin by her cardiologist during this period. CT of her chest, abdomen, and pelvis on February 10, 2021 did not reveal any signs of disease progression and demonstrated normal splenic artery anatomy (Figure 1).

**FIGURE 2:**
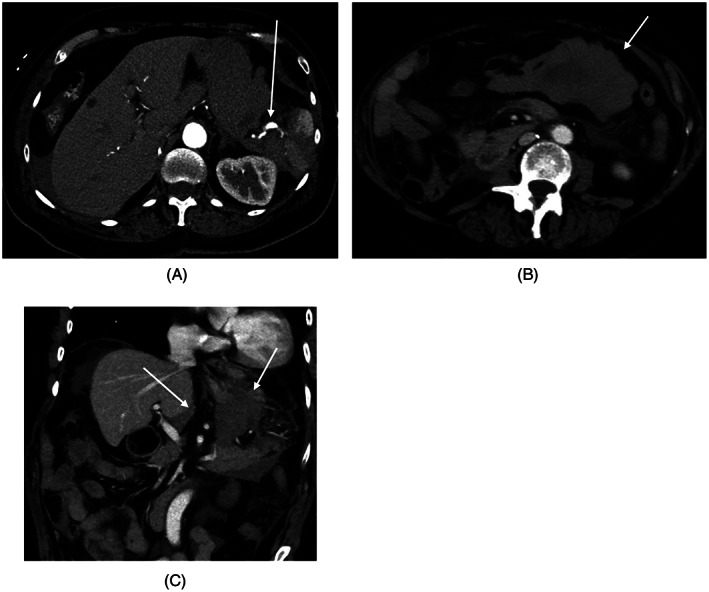
CT SCAN with contrast acquired March, 2020 to investigate patient's left upper quadrant pain: (A) Axial CT SCAN shows ruptured splenic artery aneurysm (arrow) with perisplenic hematoma and hemoperitoneum; (B) Axial CT SCAN shows hemoperitoneum in the lesser sac (arrow) due to ruptured splenic artery aneurysm; (C) Coronal CT SCAN shows hemoperitoneum in the lesser sac (arrow) due to ruptured splenic artery aneurysm

On March 5, 2021 at 10 pm, the patient suddenly developed excruciating, sharp left upper quadrant pain that radiated to her entire abdomen. She had associated nausea and loose bowel movements but no vomiting, fever, lightheadedness, rashes or petechiae. She activated emergency medical services and was brought to her local emergency department in Northern Ontario. Her pain improved with IV opioid analgesics, but her abdomen became continually distended and her hemoglobin dropped into the low 70s. A CT of her abdomen and pelvis was performed to reveal free fluid within the peritoneal cavity and contrast pooling noted adjacent the splenic hilum, related to the splenic artery (Figure 2). The splenic artery measured 1.5 cm representing either a ruptured aneurysm or pseudoaneurysm. The patient ultimately received Vitamin K, 6 units of red blood cells and was transferred to a tertiary center elsewhere in Ontario for definitive treatment of her ruptured splenic artery aneurysm.

**FIGURE 3:**
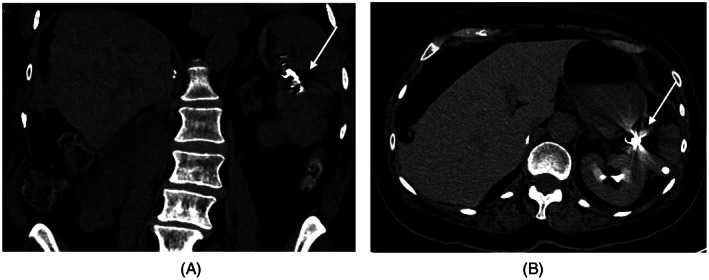
CT SCAN images were obtained post coiling (arrow) of the ruptured splenic artery aneurysm (A) Coronal image; (B) Axial image

She had coil embolization of the splenic artery by intervention radiology (Figure 3). Her recovery was complicated by a post‐operative hematoma and elevated troponin, no intervention was required.

After an extended stay in hospital, the patient was discharged home and pazopanib was discontinued. In a follow‐up appointment, the patient does not disclose any complaints and states her home blood pressure has been stable. The patient remains in good health at the time of writing this case report.

## 3 DISCUSSION/CONCLUSION

This case report outlines the development of a splenic arterial aneurysm and subsequent rupture in a patient being treated with pazopanib for metastatic renal cell carcinoma. This normotensive patient had minimal cardiovascular risk factors before starting pazopanib which included a remote 3 pack year smoking history and a family history of a father who died from myocardial infarction in his 60s. She developed hypertension within months of starting pazopanib and developed atrial fibrillation in early 2021 but had no other cardiovascular adverse events after 5 years on treatment. As a vascular endothelial growth factor inhibitor, pazopanib is commonly known to be associated with reversible hypertension (40%) and left ventricular dysfunction but the incidence of bleeding (9%–14%), serious bleeding events (6%–7%), and thromboembolic events (<1%) are rare occurrences for pazopanib within the literature.^2^, ^3^ Even more rare are the case reports of arterial aneurysms and ruptures amongst VEGF inhibitors overall. There are no case reports of such events being associated with pazopanib to the best of our knowledge.

This patient's case is interesting because of her long cumulative exposure to pazopanib. Funahashi et al. reported a case of fatal aortic dissection in a patient being treated with pazopanib and lapatinib followed by a course of sunitinib, all over the course of 5 years.^7^ This highlights the importance of monitoring patients for long‐term toxicities for VEGF inhibitors. Interestingly, in Funahashi's case the patient's VEGF inhibitors were also held briefly due to intolerance issues and then their patient's arterial rupture occurred within weeks of restarting. Our patient had normal splenic artery anatomy by CT on February 10 (Figure 1), restarted her pazopanib on February 22 and then her splenic artery had developed an aneurysm and rupture all by March 5. These events do not seem to be common but perhaps closer monitoring is required within the immediate period of restarting these medications with low thresholds for ordering imaging studies.

Pazopanib has been shown to be an effective targeted therapy for patients with metastatic renal cell carcinoma but its cardiovascular side effects can be life threatening if not monitored closely. Adequate monitoring should be performed for patients taking VEGF inhibitors, even those with minimal risk factors or long histories of drug tolerance. Patients should be informed to have low thresholds for reporting symptoms and we as clinicians should have low thresholds to initiate investigations.

## CONFLICT OF INTEREST

The authors have stated explicitly that there are no conflicts of interest in connection with this article.

## AUTHOR CONTRIBUTIONS

All authors had full access to the data in the study and take responsibility for the integrity of the data and the accuracy of the data analysis. *Conceptualization*, M.F.K.I.; *Methodology*, D.T., M.F.K.I.; *Writing—Original Draft*, D.T.; *Writing—Review & Editing*, D.T., A.A., M.F.K.I.; *Supervision*, M.F.K.I.; *Project Administration*, M.F.K.I.

## ETHICAL STATEMENT

This report was waived for review by the Thunder Bay Regional Health Sciences Centre Ethics Review Board. Formal decision on August 19, 2021. Written informed consent was obtained from the patient to publish the above information.

## Data Availability

The data that support the findings of this study are available from the corresponding author upon reasonable request.
